# Impact of glycogen storage disease type I on adult daily life: a survey

**DOI:** 10.1186/s13023-021-02006-w

**Published:** 2021-09-03

**Authors:** Sven F. Garbade, Viviane Ederer, Peter Burgard, Udo Wendel, Ute Spiekerkoetter, Dorothea Haas, Sarah C. Grünert

**Affiliations:** 1 Division of Pediatric Neurology and Metabolic Medicine, Center for Pediatric and Adolescent Medicine, University Hospital Heidelberg, Heidelberg, Germany; 2 Department of General Pediatrics, Adolescent Medicine and Neonatology, Medical Center, University of Freiburg, Faculty of Medicine, Mathildenstraße 1, 79106 Freiburg, Germany; 3 Medical Faculty of the Heinrich-Heine University Düsseldorf, Düsseldorf, Germany

**Keywords:** Glycogen storage disease type I, Glucose-6-phosphatase, Glucose-6-phosphate transporter, Coping, Quality of life, Disease burden

## Abstract

**Background:**

Glycogen storage disease type I (GSD I) is a rare autosomal recessive disorder of carbohydate metabolism characterized by recurrent hypoglycaemia and hepatomegaly. Management of GSD I is demanding and comprises a diet with defined carbohydrate intake and the use of complex carbohydrates, nocturnal tube feeding or night-time uncooked cornstarch intake, regular blood glucose monitoring and the handling of emergency situations. With improved treatment, most patients nowadays survive into adulthood. Little research has been performed on the impact of GSD I on daily life, especially in adult patients.

**Results:**

In this multi-centre study we assessed the impact of GSD I on adult daily life in 34 GSD I patients (27 GSD Ia, 7 GSD Ib) between 17 and 54 years (median 26 years) using a self-designed questionnaire that specifically focused on different aspects of daily life, such as job situation, social life, sports, travelling, composition of the household, night-time and day-time dietary management and disease monitoring as well as the patient’s attitude towards the disease. At the time of investigation, the majority of patients either attended school or university or were employed, while 3 patients (9%) were out of work. Most patients ranked GSD I as a disease with moderate severity and disease burden. Dietary treatment was considered challenging by many, but the vast majority of patients considered life with GSD I as well-manageable.

**Conclusions:**

Although the management of GSD I poses a significant burden on daily life, most patients live an independent adult life, have a positive attitude towards their disease and seem to cope well with their situation.

**Supplementary Information:**

The online version contains supplementary material available at 10.1186/s13023-021-02006-w.

## Background

Glycogen storage disease type I (GSD I, OMIM 613742) is a rare autosomal recessive disorder of carbohydate metabolism. Two subtypes are clinically and genetically distinguished: GSD Ia is caused by variations in *G6PC* resulting in deficiency of glucose-6-phosphatase (G6P), while GSD Ib is due to deficiency of the glucose-6-phophate transporter in the endoplasmatic reticulum, encoded by *SLC37A4* [1, 5]. GSD Ia/b are the most severe forms among hepatic GSDs, as G6P and the glucose-6-phosphate transporter are involved in both glycogenolysis and gluconeogenesis. The prevalence is approximately 1:100,000, with GSD Ia accounting for about 80% of cases [1].

GSD Ia is clinically characterised by severe fasting hypoglycaemia, hepatomegaly, failure to thrive, growth retardation, short stature, truncal obesity, doll-like facies, bleeding tendency, and hypotrophic muscles [1]. Laboratory findings include hyperuricemia, hyperlipidemia, and elevated lactate concentrations. Additionally, GSD Ib is associated with neutropenia and neutrophil dysfunction resulting in frequent and often severe bacterial infections and possible chronic inflammatory bowel disease [5]. Patients with GSD I generally appear normal at birth and usually present in infancy or early childhood. Treatment aims to prevent hypoglycaemia, thereby minimizing the secondary metabolic derangements and clinical symptoms. This requires regular meals with a defined carbohydrate intake and the use of complex carbohydrates. Fasting tolerance is significantly reduced but variable among patients and can improve with age. Nocturnal management is essential, either by continuous gastric tube feeding of carbohydrates or—depending on the age of the child and patient’s/family’s preference—by intake of calculated amounts of slowly resorbing uncooked cornstarch or Glycosade®, a hydrothermally treated starch with a high amylopectin content [5]. As patients are prone to hypoglycemic events, they usually have an emergency protocol, a sick-day regimen to prevent hypoglycaemia during intercurrent illnesses, and are trained to use specific measures before high-energy demanding physical activities. With optimal metabolic control, the hepatomegaly improves and growth normalizes [5]. The frequency of long-term complications such as hepatic adenomas, osteoporosis, focal segmental glomerulosclerosis, and small fiber neuropathy has markedly decreased with improvements in therapy and good metabolic control [5].

Life expectancy in GSD I is still unknown [21]. Prior to effective treatment most patients with GSDI died during childhood, some received a liver transplantation. Nowadays, with improved treatment, most patients survive into adulthood [16]. This requires not only that patients integrate treatment and management into activities of daily life like schooling and university training, professional training, work, social activities, sports, or travelling, but also to solve developmental tasks of adulthood including autonomy, romantic relationships, sexuality and family planning, and development of an attitude towards their condition. Medical conditions, particularly those requiring strict adherence to treatment recommendations or dietary restrictions, can be associated with impaired quality of live (QoL) and emotional functioning [2, 6, 8, 11, 13, 23, 26]. Only few studies have addressed the QoL of patients with GSD I so far [12, 21, 25], particularly data on adults are scarce. This has prompted us to assess the impact of GSD I on adult life in a study sample of 34 GSD Ia and Ib patients treated in different German metabolic centres.

## Results

Thirty-four adult patients with GSD Ia (n = 27) and GSD Ib (n = 7) were enrolled in the study. The median age of patients was 26 years (range 17–54 years). About 60% of the patients were male. Characteristics of the study participants are given in Table 1. None of the patients has received liver transplantation.

**Table 1:** Characteristics of the study participants

	GSD Ia and GSD Ib	GSD Ia	GSD Ib
Number of patients	34	27 (79.4%)	7 (20.6%)
Median age (range) in years	26 (17–54)	27 (17–54)	23 (17–30)
Male	20/33 (60.6%)	15/26 (57.7%)	5 (71.4%)
Female	13/33 (39.4%)	11/26 (42.3%)	2 (28.6%)

### Living situation

Of the 34 patients, 29.4% (n = 10) reported to live alone while 32.4% (n = 11) lived with their parents or at least one parent, 20.6% (n = 7) lived together with their partner, and 17.7% (n = 6) in a flat-sharing community.

### Educational and professional status

**Fig. 1:**
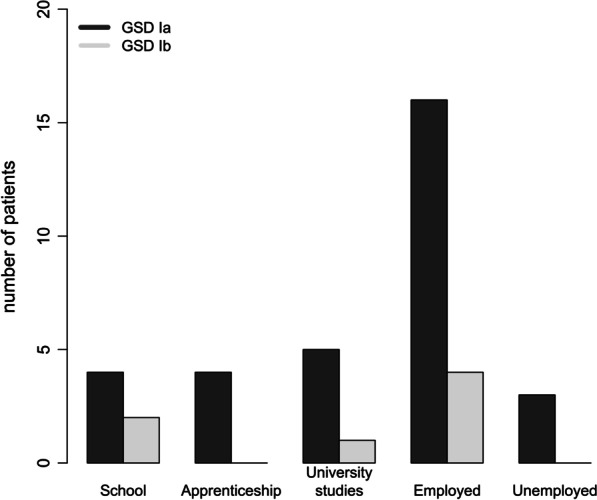
Education and professional life of 34 adult GSD I patients. The majority of patients either attended school or university or had a normal working life, while only 3 patients (9%) were unemployed

Educational and professional status are displayed in Fig. 1. The majority of patients either attended school or university or had a regular working life, while only 3 patients (9%) were unemployed. Among the working individuals, four patients (4/25; 16%) reported to work mainly physically, while 17/25 (68%) predominantly performed office work.

### Dietary management and preparation of the diet

The majority of patients (25/33; 75.8%) reported being responsible for the preparation of meals and the overall dietary management. In about one quarter of patients (8/33; 24.2%), the preparation of meals was still done by the patients’ parents. This was especially the case in younger patients: the median age of patients whose meals were prepared by their parents was 22 years (IQR 17.8–24.3 years), whereas the median age of patients preparing their meals themselves was 28 years (IQR 23–30 years, *p* = 0.024, asymptotic Mann–Whitney test). While 21.2% (7/33) of patients reported to consume only self-prepared meals during the school or working day, 39.9% (13/33) and 24.2% (8/33) also ate at a canteen or restaurant, respectively.

Most patients (21/31; 67.7%) followed a dietary regimen with a defined amount of carbohydrates per hour, but were flexible in their choice of foods and carbohydrate sources to meet the dietary requirements. Fourteen of 34 patients (41.2%) had continuous nocturnal feeds, either by a nasogastric tube (11/34; 32.4) or a percutaneous endoscopic gastrostomy (PEG) tube (3/34; 8.8%), while 15/34 (44.1%) and 11/34 (32.4%) used uncooked corn starch or Glycosade® respectively. Half of the patients on continuous nocturnal feeds (7/14; 50%) reported to flexibly switch to corn starch or Glycosade during weekends, holidays or overnight stay outside their usual environment. Twenty-eight of 31 patients (90.3%) reported beeing responsible for their nocturnal dietary management, while 3 patients (3/31; 9.7%) received support by parents or partners. 28/32 patients (87.5%) considered their nocturnal dietary management as safe.

### Metabolic control and hypoglycaemia

While 33/34 patients (97.1%) reported to possess a glucometer, one patient (1/34; 2.9%) did not have a functional device. Twelve patients (12/34; 35.3%) always carry a glucometer outside their home, 13 (13/34; 38.2%) only at times, and 9 (9/34; 26.5%) never.

**Fig. 2:**
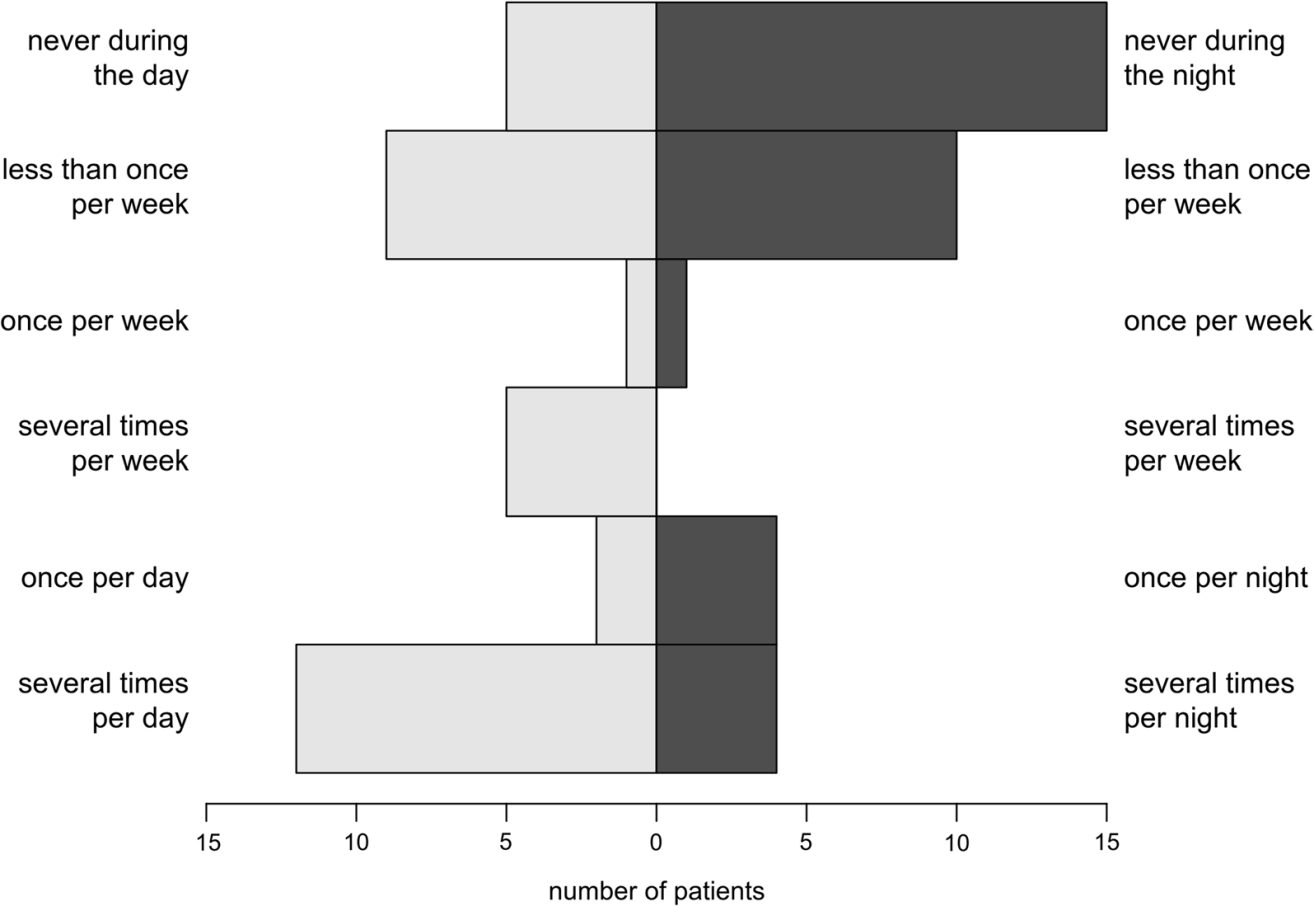
Frequency of diurnal and nocturnal blood glucose measurements in 34 adult GSD I patients

Frequencies of diurnal and nocturnal blood glucose measurements are shown in Fig. 2. Fifty percent of patients (17/34) reported always checking their blood glucose concentration when suspecting to be hypoglycemic, while 5 patients (5/34; 14.7%) never measured their blood glucose under these circumstances. The remaining 12 patients (12/34; 35.3%) only check their blood glucose level every now and then when suspecting hypoglycemia.

Twenty-five patients (25/34; 73.5%) had a continuous glucose monitoring (CGM) or at least tested a CGM device in the past. The majority of them (19/25; 76.0%) experienced CGM as helpful, while 7 patients (7/26; 26.9%) considered it not helpful. Nine patients (9/33; 27.3%) recorded their daily blood sugar profile at least every 6 months, while the majority (24/33, 72.7%) recorded blood sugar profiles less often, mostly before an appointment in the outpatient clinic. Twenty-four patients (24/34; 70.6%) reported to have had at least one diurnal hypoglycaemia within the last six months. Of these, two (8.3%) reported daily hypoglycaemias, 12 patients (50%) had one hypoglycaemia per week, six patients about one per month (24%) and 4 patients (16.7%) less than one per month. Nocturnal hypoglycaemias had occurred in 20 patients (20/33; 60.6%) within the last six months. Of these 20 patients, 13 (13/20; 65%) had at least one hypoglycaemia per month.

Episodes of severe hypoglycaemia during which patients were dependent on external help had occurred in seven patients (7/34; 20.6%) within the last 6 months.

### Visits to the outpatient clinic

Apart from 4 patients (4/34; 11.8%) of whom 2 (2/34; 5.9%) were no longer followed by a metabolic centre, all other patients were regularly seen in a metabolic outpatient clinic. The majority was followed regularly either every 6 months (12/34; 35.3%) or once per year (12/34; 35.3%). Most patients (25/31; 80.7%) attended their appointments alone, while 6 patients (6/31; 19.4%) were accompanied mainly by a parent or partner. Sixteen patients (16/30; 53.3%) expressed that they preferred to attend their medical appointments alone, while 14 (14/30; 46.7%) would be more comfortable to be accompanied. The reasons for this were diverse: Lack of confidence (1 patient), feeling more secure and comfortable (8 patients), need of transport (2 patients), out of habit (8 patients), interest of parents, partners or family members (13 patients), involvement of parents or partners in management and treatment (6 patients), to not miss important information (“four ears hear more than two”) (7 patients), and a feeling of security when the social environment is well-informed about the disease (9 patients).

Most patients (27/33; 81.8%) felt well-informed about their medical results including laboratory parameters and sonographic results, whereas 6 patients (6/33; 18.2%) denied this, mainly because the results were not explained to them by their metabolic physicians. Some patients complained that they usually do not receive the results before their next appointment in the outpatient clinic (5/21; 23.8%), or with a delay of at least one month (3/21; 14.3%).

### Physical exercise and sports

About three-quarters of patients (25/34; 73.5%) reported to exercise regularly. Most patients (28/34; 82.4%) were used to take measures to prevent hypoglycaemia during physical activity and felt safe with these measures (24/28; 85.7%). On the other hand, 4 patients (4/24; 14.3%) did not feel fully confident with their dietary measures during sports.

Five patients (5/34; 14.7%) considered GSD I to have only little impact on their physical performance, while 14 patients (14/34; 41.2%) perceived a moderate, and 15 patients (15/34; 44.1%) a high impact of GSD on their physical fitness.

### Emergency regimens

Only 10 patients (10/34; 29.4%) had a sick-day regimen that they followed at home during episodes of fever, diarrhoea or vomiting. However, 31 patients (31/33; 93.9%) had an emergency document that most of them always carried with them (25/30; 83.3%). Five patients (5/30; 16.7%) did not have an appropriate emergency card.

### Alcohol

Four patients (4/34; 11.8%) reported to not be well informed about the risks of alcohol consumption in GSD I. Five patients had no alcohol consumption at all (5/34; 14.7%). Alcoholic beverages that were regularly consumed by the remainder of patients were wine (10/32; 31.3%), beer (14/32; 43.8%), liqueurs (4/32; 12.5%), spirits (11/32; 34.4%), and alcopops (3/32; 9.4%).

### Travelling

All patients stated that they had travelled in the past. The majority (29/34; 85.3%) had good experiences while 5 patients (5/34; 14.7%) reported rather negative experiences. Most patients (29/34; 85.3%) could cope well with the efforts and challenges associated with travelling and enjoyed participating in different activities (24/34; 70.6%), felt safe with their dietary management (12/34; 35.3%) and had no hypoglycaemias (18/34; 52.9%). Patients with negative experiences stated that they considered the efforts associated with travelling proportionately too high (1/34; 2.9%), felt insecure with their dietary management (1/34; 2.9%), had hypoglycaemias (3/34; 8.8%) or could not take part in certain activities (1/45; 2.9%).

### Driving licence

Twenty-nine patients (29/33; 87.9%) reported to have a driving license, but two patients with a driving license (2/27; 7.4%) did usually not drive on their own.

### Coping with the disease

Most patients communicated their disease openly with family members (others than parents and siblings, 33/34; 97.1%), partners (19/21; 90.5%), friends (32/32; 100%), sporting comrades (13/20; 65%), teachers (6/15; 40%), colleagues at work (22/28; 78.6%), and superiors at work (23/29; 79.3%). Most of these persons were considered well-informed and competent to help in case of a hypoglycaemic event.

**Fig. 3:**
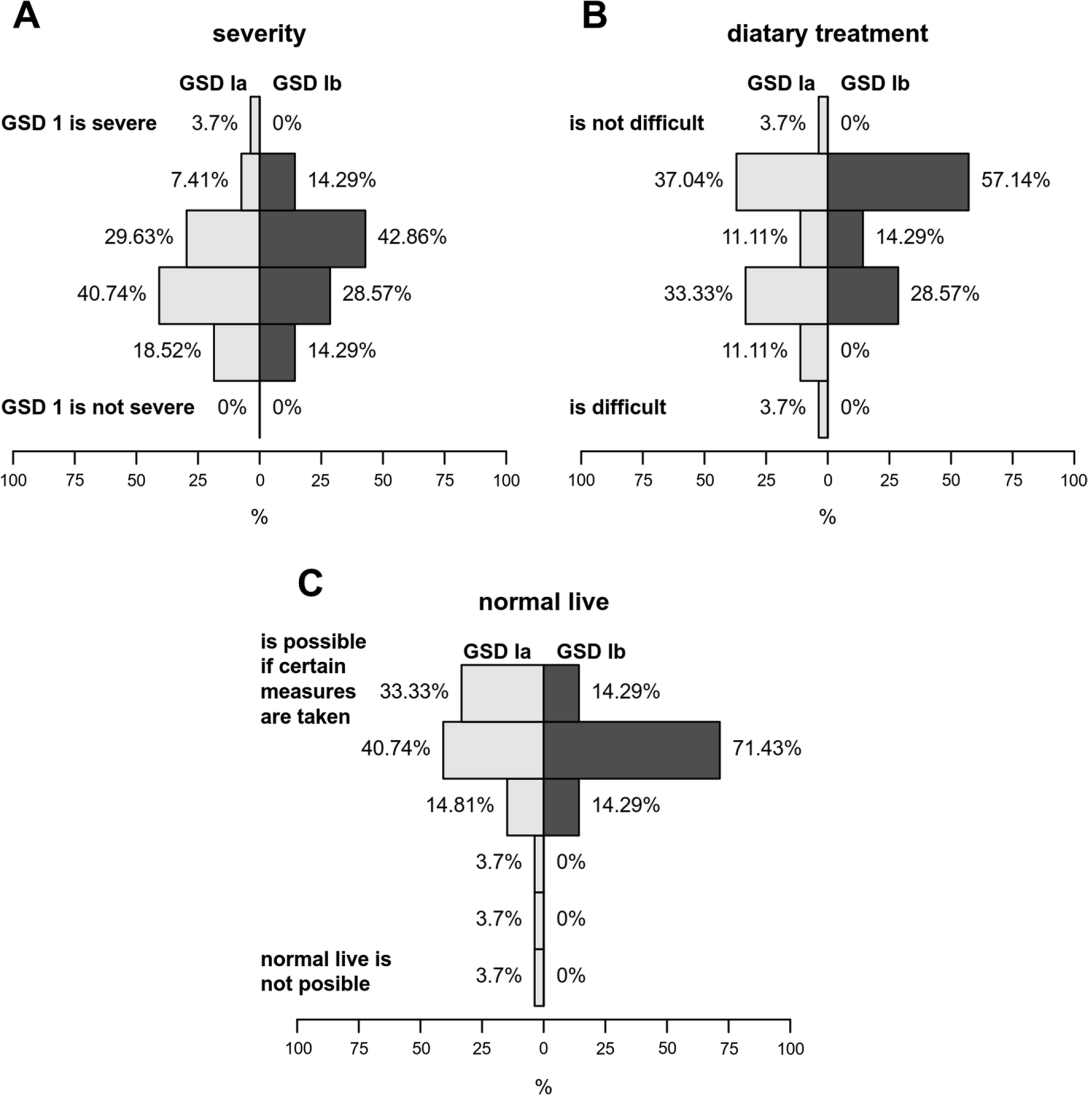
Evaluation of the severity of GSD I and the disease burden (**A**), challenges of dietary treatment (**B**), and the possibility to live a “normal life” with GSD I **(C)** (n = 34). Most patients consider GSD I a disease with moderate severity and disease burden. Attitude toward the challenges of dietary treatment was highly variable among patients. The majority of patients thought that life with GSD I is well-manageable and patients with GSD I are able to live a normal life if certain measures are taken

When asked to rate GSD I on a 6-point ordinal severity scale (1 = GSD I is no severe disorder, 6 = GSD I is a very severe disorder), most patients ranked GSD I as a disease with moderate severity and disease burden (Fig. 3A). Patients with GSD Ib perceived their disease as similarly severe as GSD Ia patients (*p* = 0.55, Mann–Whitney test; Fig. 3A). The attitude toward the challenges of dietary treatment was highly variable among patients, however many individuals reported to consider treatment as rather challenging, independent of the GSD I subtype (*p* = 0.36, Mann–Whitney test; Fig. 3B). Nevertheless, the vast majority of patients (31/34; 91.2%) thought that life with GSD I is well-manageable and patients with GSD are able to live a normal life if certain measures are taken (Fig. 3C). Again, there were no marked differences between GSD Ia and Ib patients (*p* = 0.86, Mann–Whitney test).

**Fig. 4:**
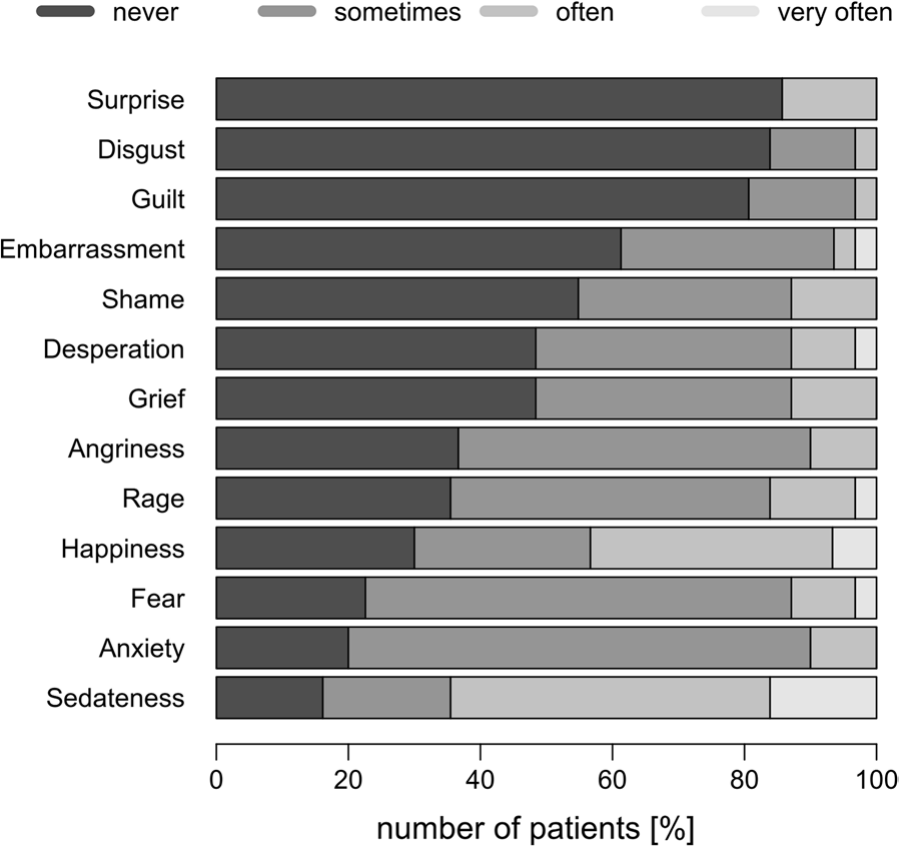
Feelings pronounced by patients in association with their disease. The most commonly mentioned negative feelings were anxiety, fear, and rage

The emotions that patients reported in association with their disease are shown in Fig. 4. The most frequently mentioned negative emotions were anxiety, fear and rage.

The impact of GSD I perceived by the patients on different aspects of adult life is shown in Table 2. Altogether, most patients had a rather positive view on their disease and their life with the disease.

**Table 2:** Perceived impact of GSD I on different aspects of adult daily life

	Perceived impact			
	Low	Moderate	High	Very high
Physical performance and fitness	5/34 (14.7%)	14/34 (41.2%)	15/34 (44.1%)	0/34 (0%)
Free time activities and friendships	21/34 (61.8%)	10/34 (29.4%)	2/34 (5.9%)	1/34 (2.9%)
Partnership	15/28 (53.6%)	10/28 (35.7%)	3/28 (10.7%)	0/28 (0%)
Financial burden (treatment costs, impact on school and professional education, career choices, choice of profession, professional life)	7/32 (21.9%)	15/32 (46.9%)	6/32 (18.8%)	4/32 (12.5%)
Sick leave at work	17/31 (54.8%)	8/31 (25.8%)	4/31 (12.9%)	2/31 (6.5%)
Intellectual performance	21/33 (63.6%)	10/33 (30.3%)	1/33 (3.0%)	1/33 (3.0%)
Emotional stability	17/33 (51.5%)	10/33 (30.3%)	4/33 (12.1%)	2/33 (6.1%)

## Discussion

Thanks to better treatment strategies the prognosis of GSD I has markedly improved within the last decades, and many patients reach adulthood without major complications. Nevertheless, GSD I remains a challenging disorder as treatment requires meticulous adherence and planning with high impact on daily life and QoL. Only little research has been performed on this topic in the past. This is especially true for adults with this rare metabolic disorder. We herein report data on the impact of GSD I on different aspects of adult life and perceived disease burden in 34 GSD I patients.

In most previous studies, QoL has been addressed with the use of standardized questionnaires: Storch et al. investigated psychosocial functioning of children with GSD Ia and Ib [25]. The authors studied 31 children and their parents using different questionnaires that addressed QoL, loneliness, family functioning, sibling relationship quality, parental distress, parenting stress, child adaptive behaviour, and child emotional and behavioural functioning. The authors showed that both types of GSD I were associated with reduced QoL and independent functioning, elevated levels of internalizing distress and parental stress relative to healthy peers. Based on these results, Sechi et al. [21] performed an Italian multicentre study on the QoL of adult GSD I patients using the standardized questionnaire SF-36 [21]. Thirty-eight patients over 16 years (median age 26.5 years) were included in this study. Their results showed that also adult patients with GSD I may have an impaired QoL. Especially patients with GSD type Ib, women, and those with renal complications were more likely to experience a poorer QoL [21]. Although patients with GSD I had lower median scores in *general health perception* and *social functioning* when compared to normative data, they had higher median scores for *bodily pain* and *mental health* which might be explained by good coping strategies. QoL data of adolescent and adult GSD I patients are also available from the Swiss hepatic glycogen storage disease registry [12]. This registry includes 27 GSD I patients between 14 and 29 years. QoL was assessed using the SF-12 questionnaire, and in contrast to the above mentioned studies, scores in this sample were within the normal range [12]. Additionally, most patients were well integrated into social and professional life.

Flanagan et al. studied eating attitudes, eating disorder symptoms, and body image among 64 patients with GSD ranging from 7 to 52 years and found a lower body esteem in children, adolescents and adults with GSD compared to population norms [7]. Interestingly, patients reported growing acceptance of their bodies with age associated with less negative attitudes and behaviours in adulthood.

Our study addressed several aspects of normative adult life events as well as the disease burden perceived by the patients. Different from the above-mentioned studies, we used a self-developed questionnaire that was tailored specifically to the challenges and burdens associated with GSD I, including aspects such as dietary management. Overall, our data demonstrate that most adult GSD I patients live an independent adult life. Concerning education and work, all but 3 unemployed patients (9%) either attended school or university or had a regular working life. Very similar findings were reported by Sechi et al. [21] with an unemployment rate of 11.4% in 38 adult GSD I patients. Data from the Swiss GSD registry comparably showed that most patients were employed or in vocational training with no need of supporting services [12].

In our study, we did not ask for the reason for unemployment, but it is of note that in the European Study on GSD I (ESGSD I) with more than 200 GSD I patients, 11% were reported to need a special education or work, while 6% were unable to have a profession because of mental disability [19]. However, it is important to bear in mind that the ESGSD study includes patients from the “pre-cornstarch aera”, and it is well-conceivable that poorer metabolic control and also possibly later diagnosis might have contributed to a poorer neurologic outcome.

At the time of the study, most patients lived an independent life, while about 32% still lived with their parents (median age of patients living with their parents was 22 years). In this respect, GSD I patients do not seem to differ significantly from the normal population, as German demographic data show that more than 28% of 25-year-olds still live with their parents [24]. About one quarter reported that parents were still mainly responsible for the preparation of meals and dietary management. Difficulty in becoming independent from parents has been observed in patients with inherited metabolic diseases in general [14, 21]. This is well understandable considering the high level of parental involvement in disease management during infancy and childhood [14, 21]. It is also of note, that almost half of the patients in our study preferred to be accompanied to visits in the metabolic outpatient clinic. Supporting patients’ personal responsibility should be one major aim in the transition process from adolescence to adulthood. This includes the early involvement of the patient in the treatment and disease monitoring together with age-appropriate communication and information by doctors during outpatient visits. Providing appropriate information empowers the individual, giving them confidence to manage their disorder in the future [14]. Several patients in our study stated that results of outpatient visits such as laboratory values and necessary therapeutic adaptions were often not well communicated to them.

Living with a chronic disease might not only impact QoL due to the disorder itself, but also due to the necessary treatment, which may be a major challenge. For patients with GSD I this includes frequent meals, strict planning of activities, loss of spontaneity as well as sleep disturbances due to night-time interruptions for nocturnal corn starch intake [7, 20]. When asked for their opinion about the severity of GSD I, most patients ranked GSD I as a disease with moderate severity and disease burden, but rated the challenges of dietary treatment as rather high. The three negative emotions that more than 60% of patients felt with respect to their disease at least sometimes were anxiety, fear, and rage.

Among the aspects addressed in this study, the highest impact of GSD I was perceived on physical performance and fitness. More than 85% of patients either considered their physical fitness moderately or highly impaired. Additionally, some patients expressed at least some degree of uncertainty with respect to the risk of hypoglycaemia during sports. The impact of GSD I on partnership was rated low (53.6%) or moderate (35.7%) by most patients. Interestingly, Sechi et al. [21] reported a lower percentage of married patients with children in their sample of 38 Italian patients when compared to the age- and gender-matched Italian population and suggested that GSD I patients may have more difficulty in forming adult relationships and starting a family than healthy peers. Impact on free time activities and friendships was also considered low by the majority of the study patients. More than 30% of patients reported a high or very high financial impact due to their chronic disease. Studying families with a child affected by a urea cycle defect, Cederbaum et al. [4] reported financial stress as one of the greatest sources of stress in their study cohort. Financial stress affects a significant proportion of patients diagnosed with a chronic illness. In addition to costs for medication that are not all covered by insurance companies, a chronic disease may have an impact on education and professional choices, but also on the fitness for work, thereby affecting the economic status.

Overall, most patients in our study had a rather positive attitude towards their disease and felt able to live a normal life if certain measures are taken. Given the challenges and restrictions associated with GSD I this may reflect good coping strategies in most of the patients. Comparable to healthy subjects, successful coping enables individuals with a chronic illness to emphasize the positive aspects of their lives, thereby reducing general distress [3, 22]. Coping strategies are highly variable, and the perceived disease burden of an individual patient does not automatically correlate with disease severity. This is reflected by the fact that we did not observe significant differences in the perceived disease burden between patients with GSD Ia and GSD Ib, although GSD Ib in adulthood is usually associated with additional problems such as inflammatory bowel disease and other complications linked to neutropenia. Sechi et al. reported that the personal evaluation of “*general health”* given by GSD I patients was similar to that perceived by patients affected by type 2 diabetes, another chronic disease requiring a lifelong diet [15, 21].

Most patients communicated their disorder openly to family members, partners, friends, sporting comrades, teachers and colleagues. In view of the fact that GSD I can lead to life-threatening hypoglycaemia and that in these situations, patients may depend on external help, information of the patients’ social environment and competency to react properly can be lifesaving. In fact, about 20% of patients at least had one severe hypoglycaemia within the last six months during which they required external help.

One might object that our study lacks normative data from healthy subjects and the sample possibly has selection bias only including individuals successfully coping with their condition. However, results clearly vary in all items and clearly demonstrate that participants are neither perfectly compliant nor a selection of relatively mild forms. Our aim was not to do a normative comparison with healthy adults, but to explore how disease-specific facets of GSD type I interfere with adult normative life-events and developmental tasks [17]. We see the significant strength of our study, that instead of using a standardised generic questionaire, describes the QoL of adults with GSD I in a way unfolding how they struggle and cope with their condition and how they live (day and night), thereby providing essential information for all disciplines of the treatment team. Our data also provide a basis for the development of a transition program for adolescents with GSD I that covers all relevant aspects adult life. The workshop based on the items of the questionnaire allows to postulate face validity, comprehensiveness, and comprehensibility of the questionnaire (see methods paragraph). Apart from linking the questionnaire to the theory of developmental tasks we do not postulate any theoretical construct why construct, convergent and discriminant validity is not claimed. However, in further studies the questionnaire can be linked to objective measures like long-term blood glucose concentrations or physical fitness. Participants also reported behaviour not recommended for individuals with GSD I (alcohol consumption) why social desirability may not be a critical issue in our data. A limitation can be raised regarding the representativeness of our sample. Members of patient organisations may be more active copers of their condition, but on the other hand non-members may feel sufficiently competent to master their condition alone.

## Conclusions

Our study demonstrated that although GSD I is a severe disease that requires lifelong therapy with strict adherence, most patients live an independent adult life and cope well with their situation. Physicians involved in transition of GSD patients should support their patients in becoming autonomous as early as possible and address important topics such as medical monitoring, the risk of alcohol consumption, and family planning with their patients. Patient organisations that enable exchange with peers of the same age may not only contribute to better information of patients, but also provide emotional and psychosocial support.

## Methods

A questionnaire was designed by two of the authors (UW, paediatrician and PB, psychologist), both having long-lasting clinical experience in treatment and care of individuals with GSD I as well as patient workshops dealing with self-mangement and coping with the condition, to address important aspects of daily life with GSD I in adulthood. Item construction followed Havidhurst’s theory of developmental tasks [9] originating from biology (e.g. physical changes and health related issues), the self (achieving emotional and practical everyday independence), and social expectations (preparing for a professional economic career, achieving sexual and romantic relations). The items cover school and professional education, the job situation, social life, sports, travelling, composition of the household, dietary management and disease monitoring as well as the patient’s attitude towards his/her disease. Pre-diagnosed psychological conditions such as anxiety or depression were not assessed by the questionnaire. An English translation of the questionnaire can be found in the Additional file 1: Supplemental Material. For this study, subjects were recruited on the occasion of a workshop held at the Annual meeting of the German patient organisation for glycogen storage diseases (Duderstadt 2017) and via the Metabolic Centres Freiburg and Heidelberg. The workshop was divided in two parts. In part one participants filled in the pseudonymized questionnaire, in part two participants shared their experience with particular items of the questionnaire (e.g. travelling, night-time feeding). During the workshop no further issues were introduced and no difficulties of item comprehensibility was reported. The entire study population is referred to as “adults”, although two 17 year-old individuals were included. The study was approved by the ethics committees of the universities Freiburg and Heidelberg (EKFR Nr. 468/18, S-022/2019).

### Statistical analysis

Data analysis was performed using the Software R (https://www.r-project.org) [18]. Descriptive and explorative analysis was used to describe the study sample. Continuous data is reported with mean and standard deviation, count data is presented as frequencies and percentages. No a-priori hypotheses are tested. We used asymptotic Mann–Whitney Test from R package ‘coin’ to compare medians between two groups [10].

## Supplementary Information

**Additional file 1. English translation of the questionnaire used in this study to assess the impact of GSD I on adult daily life.**

## Data Availability

The datasets used and/or analysed during the current study are available from the corresponding author on reasonable request.

## Abbreviations

CGM: Continuous glucose monitoring
ESGSD: European study on GSD I
GSD I: Glycogen storage disease type I
G6P: Glucose-6-phosphatase
IQR: Interquartile range
PEG: Percutaneous endoscopic gastrostomy
QoL: Quality of Life
