# Supplementary material for: Impact of glycogen storage disease type I on adult daily life: a survey
Source: Orphanet J Rare Dis. 2021 Sep 3;16:371. doi: 10.1186/s13023-021-02006-w (PMC8414849; doi:10.1186/s13023-021-02006-w)
Supplement: Supplementary file 1 — Additional file 1. English translation of the questionnaire used in this study to assess the impact of GSD I on adult daily life. [file 13023_2021_2006_MOESM1_ESM.docx]

Dear GSD patient!

We invite you to participate in a survey on the impact of GSD on daily life. All data will be processed pseudonymously, which means that a code is used instead of your name or any personal data by which you could be identified.

If you wish to have access to your personal data later, please add a personal code on the first page.

This questionnaire has been developed for adult patients with glycogen storage disease type I (GSD I). We are interested to learn how you carry out your treatment and which problems you are faced with. With your contribution to this survey you can draw our attention to aspects that might be important for the development of a training program for adolescents and young adults with GSD I.

Thank you very much for your participation and contribution!

Prof. Dr. med. Udo Wendel PD Dr. phil. Peter Burgard

***Instruction***

*PLEASE CONSIDER THE FOLLOWING RULES WHEN FILLLING IN THIS QUESTIONNAIRE*

1. *Please always tick the most appropriate answer.*
2. *For some questions multiple answers can be chosen. If more than one answer applies, please tick all relevant boxes.*
3. *For some questions you are asked to write your answer as free text.*
4. *Please fill in the questionnaire completely.*

Date _ _ /_ _ /_ _ _ _ (DD/MM/YYYY)

Age years

Sex ☐ female ☐ male

1. Which type of GSD I do you have?

| GSD Ia |  |  | GSD Ib |  |
| --- | --- | --- | --- | --- |

2. What is your current living situation?

| a) I live alone |  |
| --- | --- |
| b) together with parents or one parent |  |
| c) separately from my parents, but in the same house |  |
| d) in a shared flat/house with other people |  |
| e) together with my partner |  |
| f) Other: |  |

*If you ticked „Other“, please describe.*

3. Current education/ employment

*Multiple answers possible*

| a) School |  |
| --- | --- |
| b) Vocational training/ apprenticeship |  |
| c) University studies |  |
| d) Employment |  |
| e) Unemployed |  |

4. If you are employed, what is your profession?

5. Do you mainly work physically?

☐ yes ☐ no

6. Do you mainly do office work?

☐ yes ☐ no

7. Who prepares your meals at home?

| a) Mother/ parents |  |
| --- | --- |
| b) I myself |  |
| c) I myself together with my flatmates |  |
| d) I myself together with my partner |  |
| e) Someone else: |  |

*If your answer is „Someone else“, please give details.*

8. What/Where do you eat at work/school/university?

*Multiple answers are possible.*

| a) Exclusively home-made food that I bring from home |  |
| --- | --- |
| b) Partially home-made food that I bring from home |  |
| c) Canteen |  |
| d) Restaurant |  |
| e) Other: |  |

*If you ticked „Other“, please describe.*

**Dietary management during the day**

9. Do you stick to a special dietary recommendation/prescription during the day?

| a) Yes, very strictly |  |
| --- | --- |
| b) Yes, with exceptions |  |
| c) I regularly stick to my dietary prescriptions |  |
| d) I unregularly stick to my dietary prescriptions |  |
| e) I do not follow any dietary prescription |  |

10. Do you freely exchange different foods according to the prescribed carbohydrate amount in your dietary prescription?

☐ yes ☐ no

10.1 If „yes “, do you do this on your own or are you supported in exchanging foods?

| a) I do this on my own |  |
| --- | --- |
| b) I am supported by my mother/ my parents |  |
| c) I am supported by other family members |  |
| d) I am supported by flatmates |  |
| e) I am supported by my partner |  |
| f) I am supported by someone else: |  |

*Please specify if you are supported by someone else.*

**Nighttime management**

11. What kind of a regimen do you follow at night?

*Multiple answers are possible.*

| a) continuous drip feeding via a nasogastric tube |  |
| --- | --- |
| b) continuous drip feeding via PEG tube |  |
| c) with uncooked cornstarch |  |
| d) with Glykosade® |  |
| f) I change from **a or b** to **c or d** for special occasions (weekends, overnight stays, travelling/ holidays) |  |
| e) I have no nocturnal nutrition |  |

11.1 If you use uncooked cornstarch or Glykosade® at night, please give details on time and amount.

| **Uncooked cornstarch** | |  | **Glykosade®** | |
| --- | --- | --- | --- | --- |
| Time | Amount in grams |  | Time | Amount in grams |
|  |  |  |  |  |
|  |  |  |  |  |
|  |  |  |  |  |
|  |  |  |  |  |

12. Have you changed your nighttime regimen after achieving adult age?

☐ yes ☐ no

12.1 If „yes“, what kind of regimen did you follow previously?

| a) continuous drip feeding via a nasogastric tube |  |
| --- | --- |
| b) continuous drip feeding via PEG tube |  |
| c) with uncooked cornstarch |  |
| d) with Glykosade® |  |
| f) I change from **a or b** to **c or d** for special occasions (weekends, overnight stays, travelling/ holidays) |  |
| e) I had no nocturnal nutrition |  |

12.2 If you have changed your dietary regimen after the age of 18, what was the reason for this?

13. Do you take care of your nighttime management alone?

☐ yes ☐ no

13.1 If „no“, who assists you with which task?

| **Task** | **Assisted by** |
| --- | --- |
| a) Preparation of maltodextrin solution |  |
| b) Preparation of the amount of cornstarch or Glycosade |  |
| c) Waking up for taking cornstarch or Glycosade |  |
| d) Blood glucose measurements |  |

14. Do you consider your nighttime regimen as safe?

☐ yes ☐ no

14.1 If „no“, why not?

**Blood glucose measurements**

15. Do you have a functional glucometer?

☐ yes ☐ no

16. When you leave your house for a while do you carry your glucometer with you?

| a) Yes, always |  |
| --- | --- |
| b) Sometimes |  |
| c) No, never |  |

17. How often do your measure your blood glucose concentration **during the day**?

| a) Several times per day |  |
| --- | --- |
| b) Once per day |  |
| c) Several times per week |  |
| d) Once per week |  |
| e) Less than once per week |  |
| f) I don’t check my blood glucose during the day |  |

18. How often do your measure your blood glucose concentration **during the night**?

| a) Several times per night |  |
| --- | --- |
| b) Once per night |  |
| c) Several times per week |  |
| d) Once per week |  |
| e) Less than once per week |  |
| f) I don’t check my blood glucose during the night |  |

19. Do you check your blood glucose when you assume being hypoglycemic?

| a) Yes, always |  |
| --- | --- |
| b) Sometimes |  |
| c) No, never |  |

20. How often do you record a 24h blood glucose profile?

| a) Weekly |  |
| --- | --- |
| b) Monthly |  |
| c) Every 3 months |  |
| d) Every 6 months |  |
| e) Less frequently than every 6 months |  |
| f) Sporadically |  |
| g) Only before a visit to the outpatient clinic |  |

21. Have you ever performed **continuous glucose monitoring**?

☐ yes ☐ no

21.1 Did you find continuous glucose monitoring helpful?

☐ yes ☐ no

**Hypoglycemia**

22. Have you had at least one hypoglycemia **during the day** within the last 6 months?

☐ yes ☐ no

22.1 If „yes“, how often did you have hypoglycemias during the day?

| a) Several times a day |  |
| --- | --- |
| b) Daily |  |
| c) Weekly |  |
| d) Once per month |  |
| e) Less than once per month |  |

23. Have you had at least one hypoglycemia **during the night** within the last 6 months?

☐ yes ☐ no

23.1 If „yes“, how often did you have hypoglycemias during the night?

| a) Several times a day |  |
| --- | --- |
| b) Daily |  |
| c) Weekly |  |
| d) Once a month |  |
| e) Less than once a month |  |

24. Did you have one or more severe hypoglycemias during the last 2 years during which you were dependent on external help?

☐ yes ☐ no

24.1 If „ yes“, how often?

a.) during the day times

b.) during the night times

**Outpatient visits**

25. How often did you attend a metabolic outpatient clinic within the last 2 years?

| a) Regularly, more frequently than every 3 months |  |
| --- | --- |
| b) Regularly every 3 months |  |
| c) Regularly every 6 months |  |
| d) Regularly once a year |  |
| e) Regularly less than once a year |  |
| f) Irregularly |  |
| g) Only after invitation by the metabolic outpatient department |  |
| h) Never |  |

26. Do you usually come to the metabolic outpatient department…

☐ alone ☐ accompanied

26.1 If „accompanied“, who accompanies you?

| a) Parent or parents |  |
| --- | --- |
| b) Someone else from your family |  |
| c) Partner |  |
| d) Friend |  |
| e) Someone else: |  |

*If „Someone else“ please specify who accompanies you.*

27. If you could choose, would you prefer to come to the outpatient clinic alone or accompanied?

| a) Alone |  |
| --- | --- |
| b) Accompanied |  |

27.1 If you prefer to be accompanied , what are the reasons for that?

Several answers are possible.

| a) Lack of self-confidence |  |
| --- | --- |
| b) I feel safer when accompanied |  |
| c) I depend on help for transport |  |
| d) Out of habit |  |
| e) My parents/my partner are interested |  |
| f) The persons who accompany me are involved in my treatment |  |
| g) Four ears hear more than two |  |
| h) I feel safer if my parents/my partner are informed about all aspects of my GSD |  |

28. Do you feel well-informed about your current examination results (laboratory investigations, ultrasound)?

☐ yes ☐ no

28.1 If „no“, why not?

| a) I was not informed about the results |  |
| --- | --- |
| b) The results were not explained to me |  |
| c) The results were communicated to me, but I don’t understand them |  |
| d) I don’t care about the results |  |

29. How long does it usually take until you receive the results of your outpatient visit?

| a) Less than one week after the outpatient visit |  |
| --- | --- |
| b) Less than two weeks after the outpatient visit |  |
| c) Less than one month after the outpatient visit |  |
| d) It takes more than one month after the outpatient visit |  |
| e) I never get the results until the next visit to the outpatient clinic |  |

**Sports and physical excercise**

30. Do you regularly excercise?

☐ yes ☐ no

31. Do you take precautions to prevent hypoglycemia during exercise (sports, physical activity or dancing in a disco)?

☐ yes ☐ no

31.1 If „no“, why not?

| a) I do not perform physical excercise. |  |
| --- | --- |
| b) I am not sufficiently informed what to do. |  |
| c) I have not had any hypoglycemias in the past even without taking precautions. |  |
| d) I haven’t cared about it yet. |  |
| d) I just forgot. |  |

31.2 If „yes“, how do you feel with these precautions?

| a) Safe |  |
| --- | --- |
| b) Not safe |  |

**Sick-day management**

32. Do you use a special sick-day dietary regimen at home during febrile infections, diarrhea or vomiting?

| a) yes |  |
| --- | --- |
| b) no , but I have a sick-day dietary regimen |  |
| c) no , I don’t have a sick-day dietary regimen |  |

33. Do you have an emergency card?

☐ yes ☐ no

33.1 If „yes“, do you always carry it with you?

| a) yes |  |
| --- | --- |
| b) no, I don’t carry it with me. |  |

33.2. If „no“, why not?

34. Have you ever slept outside home?

☐ yes ☐ no

34.1. If „no“: Are there reasons preventing you from overnight stays?

Several answers are possible

| a) I just haven’t had the chance yet |  |
| --- | --- |
| b) I shy away from the efforts associated with my GSD |  |
| c) I feel unsafe due to my GSD |  |
| d) My parents/partner/family members are against it |  |

**Alcohol consumption**

35. Do you know the risks of alcohol consumption in GSDs?

☐ yes ☐ no

35.1. If „no“, why not?

| a) I never drink alcohol. |  |
| --- | --- |
| b) I have never been told about the risks of alcohol consumption. |  |
| c) I haven’t cared about it in the past. |  |

35.2 Which of the following alcoholic beverages do you consume?

| a) Wine |  |
| --- | --- |
| b) Beer |  |
| c) Liqueurs |  |
| d) Spirits (Cognac, Whiskey, Rum, Vodka) |  |
| e) Alcopops |  |

**Travelling**

36. Have you ever travelled?

☐ yes ☐ no

36.1 If „yes“, which experiences have you made?

*Multiple answers may apply*

| a) **Good**. The efforts were well-manageable. |  |
| --- | --- |
| b) **Good**. I felt safe with my dietary regimen. |  |
| c) **Good**. I didn’t have hypoglycemias. |  |
| d) **Good**. I could take part in all activities and made good experiences. |  |
| e) **Bad**. The efforts associated with my GSD were too big. |  |
| f) **Bad**. I felt unsafe, especially with my dietary regimen. |  |
| g) **Bad**. I had hypoglycemias. |  |
| f) **Bad**. I could hardly take part in activities. |  |

36.2. If „no“, are there reasons why you haven’t travelled so far?

*Several answers may apply*

| a) I just haven’t had the chance yet. |  |
| --- | --- |
| b) I shy away from the efforts associated with my GSD |  |
| c) I feel unsafe due to my GSD |  |
| d) My parents/partner/family members are against it |  |

37. Do you have a driving licence? (Car or motorbike)

☐ yes ☐ no

37.1 If „yes“, do you drive on your own?

☐ yes ☐ no

**Persons in your social environment**

38. Do persons in your social environment know about your GSD?

| *Several answers may apply* | yes | no |
| --- | --- | --- |
| a) Family members apart from parents/siblings |  |  |
| b) Partner |  |  |
| c) Friends |  |  |
| d) Sporting comrades |  |  |
| e) Teachers |  |  |
| f) Colleagues |  |  |
| g) Superiors at work |  |  |
| h) Others |  |  |

If you ticked „others“, please specify.

39. Are the persons in your social environment well-informed enough about your GSD that they could help you in case of hypoglycemia?

| *Multiple answers are possible* | yes | no |
| --- | --- | --- |
| a) Family members apart from parents/siblings |  |  |
| b) Partner |  |  |
| c) Friends |  |  |
| d) Sporting comrades |  |  |
| e) Teachers |  |  |
| f) Colleagues |  |  |
| g) Superiors at work |  |  |
| h) Others |  |  |

If you ticked „others“, please specify.

40. How often do you think about your GSD?

| a) Several times a day |  |
| --- | --- |
| b) Daily |  |
| c) At least once a week |  |
| d) Once a month or less often |  |
| e) Only when I have an appointment in the outpatient clinic |  |

41. Please rate the severity of GSD I on the scale.

| GSD I is **not** a severe disease | **1** | **2** | **3** | **4** | **5** | **6** | GSD I **is** a  severe disease |
| --- | --- | --- | --- | --- | --- | --- | --- |
|  |  |  |  |  |  |  |  |

42. Please give your opinion with respect to the challenges of GSD treatment.

| I struggle with the management/treatment of my disease | **1** | **2** | **3** | **4** | **5** | **6** | I don’t have any problems with the management/treatment of my disease |
| --- | --- | --- | --- | --- | --- | --- | --- |
|  |  |  |  |  |  |  |  |

43. Please give your personal opinion on the following two statements:

| With GSD I normal life is not possible. | **1** | **2** | **3** | **4** | **5** | **6** | If certain measures are taken normal life is possible with GSD I |
| --- | --- | --- | --- | --- | --- | --- | --- |
|  |  |  |  |  |  |  |  |

**Impact of GSD I on your daily life**

44. Please rate the impact of your GSD on different aspects of daily life

|  | low | moderate | high | very high |
| --- | --- | --- | --- | --- |
| physical activity/ excercise capacity |  |  |  |  |
| freetime activities, friedships, partnerships |  |  |  |  |
| economic/financial issues, i.e. treatment costs, impact on schooling and professional choices, working life, sick leave etc. |  |  |  |  |
| intellectual capacity |  |  |  |  |
| emotional balance |  |  |  |  |

45. How often do you have the feelings mentioned below associated with your GSD?

|  | *Never* | *Sometimes* | *often* | *very often* |
| --- | --- | --- | --- | --- |
| Happiness |  |  |  |  |
| Rage |  |  |  |  |
| Fear |  |  |  |  |
| Disgust |  |  |  |  |
| Grief |  |  |  |  |
| Anxiety |  |  |  |  |
| Shame |  |  |  |  |
| Sedateness |  |  |  |  |
| Desperation |  |  |  |  |
| Guilt |  |  |  |  |
| Embarassment |  |  |  |  |
| Anger |  |  |  |  |
| Surprise |  |  |  |  |

46. Would you take part in an educational program for living with GSD?

☐ yes ☐ no

**Thank you for your participation!**
